# Supplementary material for: Remdesivir Decreases Mortality in COVID-19 Patients with Active Malignancy
Source: Cancers (Basel). 2022 Sep 28;14(19):4720. doi: 10.3390/cancers14194720 (PMC9563758; doi:10.3390/cancers14194720)
Supplement: Supplementary file 1 [file cancers-14-04720-s001.zip › cancers-1916222-supplementary.pdf]

Table S1. Baseline characteristics of studied cohort stratified by gender and early hospital death.

Characteristic	Died n=59		Survived n=163		P value *
Gender	Female n=20	Male n=39	Female n=87	Male n=76	0.0146
Age in years, median (IQR)	73 (68-81)	69 (63-81)	69 (62-78)	70 (65-76)	0.3105
BMI in kg/m2, median (IQR)	29.4 (25.6-32.4)	25.2 (23.0-30.3)	26.2 (23.0-29.4)	27.1 (24.0-29.4)	0.7718
Hematological cancer, n (%)	6 (30.0)	6 (15.4)	29 (33.3)	19 (25.0)	0.2310
SpO2 in % at hospital admission, median (IQR)	89 (82-95.5)	82 (74-91)	92 (89-95)	92 (87-96)	<0.0001
CRP baseline [mg/dL], median (IQR)	115.1 (39.1-146.0)	95.8 (40.7-195.0)	55.4 (24.5-90.8)	61.6 (19.7-143-7)	0.0008
Procalcitonin baseline [ng/mL], median (IQR)	0.18 (0.06-1.69)	0.29 (0.14-0.91)	0.09 (0.05-0.22)	0.18 (0.05-0.42)	0.0017
WBC baseline [/uL], median (IQR)	6620 (3015-11110)	7930 (4930-13600)	4605 (3420 -722)	6090 (3855-9200)	0.1280
Lymphocytes baseline [/uL] , median (IQR)	630 (510-1000)	700 (490-950)	960 (640-1340)	1000 (700-1400)	0.0014
Neutrocytes baseline [/uL], median (IQR)	3350 (1770-8550)	5910 (3000-11340)	2955 (1925-5140)	3900 (2440-6400)	0.0091
PLT baseline [/uL], median (IQR)	175000 (94200-251500)	155000 (121000-251000)	178500 (136000-256000)	179000 (128000-241000)	0.5771
IL-6 baseline [pg/mL], median (IQR)	77.7 (38.3-163.2)	169.3 (60.5-467.3)	41.0 (18.4-86.7)	50.1 (13.2-100.5)	<0.0001
D-dimers baseline [ug/mL], median (IQR)	1500 (1166-2290)	1940 (880-5620)	1035 (662-2167)	1215 (751-1812)	0.0025
eGFR < 60 ml/min/m^2 baseline, n (%)	10 (52.6)	21 (55.3)	21 (24.1)	24 (31.6)	0.0004
Use of other medication at baseline, n (%)	15 (75.0)	36 (92.3)	74 (87.1)	63 (82.9)	0.6666
Multimorbidity, n (%)	14 (70.0)	29 (74.4)	57 (65%)	55 (72.4)	0.6212
Use of RDV, n (%)	4 (20.0)	5 (12.8)	28 (32.2)	21 (27.6)	0.0370
Use of TCZ , n (%)	3 (15.0)	10 (25.6)	10 (11.5)	13 (17.1)	0.2150
Use of dexamethasone, n (%)	12 (60.0)	25 (64.1)	40 (46.0)	33 (43.4)	0.0226
Use of convalescent plasma, n (%)	3 (15.0)	5 (12.8)	20 (32.2)	15 (19.7)	0.2488

* P between patients who died vs who survived. * Fisher exact test; Abbreviations: BMI – body mass index; IQR - interquartile range; SpO2 - oxygen peripheral blood saturation; CRP – C-reactive protein; WBC – white blood cells, PLT – platelets; RDV – remdesivir; TCZ – tocilizumab
